# Association of Hearing Acuity and Cognitive Function Among a Low-Income Elderly Population in Rural China: A Population-Based Cross-Sectional Study

**DOI:** 10.3389/fnins.2021.704871

**Published:** 2021-08-16

**Authors:** Yi Xu, Yan Li, Dandan Guo, Xin Zhang, Huiying Guo, Hui Cao, Xin Li, Jing Zhang, Jun Tu, Jinghua Wang, Xianjia Ning, Dong Yang

**Affiliations:** ^1^Department of Otorhinolaryngology, Tianjin Medical University General Hospital, Tianjin, China; ^2^Department of Anesthesiology, Tianjin Jizhou People’s Hospital, Tianjin, China; ^3^Department of Neurology, Tianjin Medical University General Hospital, Tianjin, China; ^4^Department of Otorhinolaryngology, Beijing Tsinghua Changgung Hospital, School of Clinical Medicine, Tsinghua University, Beijing, China; ^5^Laboratory of Epidemiology, Tianjin Neurological Institute, Tianjin, China; ^6^Key Laboratory of Post-Neuroinjury Neuro-Repair and Regeneration in Central Nervous System, Tianjin Neurological Institute, Ministry of Education and Tianjin City, Tianjin, China

**Keywords:** cognitive function, hearing loss, pure tone average, low-frequency pure tone average, aging

## Abstract

Hearing loss is a modifiable risk factor for dementia and cognitive decline. However, the association between cognition and hearing acuity at different frequencies is unknown. We aimed to assess the relationships between hearing acuity at different frequencies with global cognitive function and five domains of cognition among a low-income elderly population in northern rural China. A population-based cross-sectional study was conducted to collect basic information from elderly residents aged 60 years and older in rural areas of Tianjin, China from April 2012 to November 2013. Pure tone averages (PTAs) at different frequencies in the ear with better hearing and Mini-Mental State Examination (MMSE) scores were measured, and the relationships between these variables were assessed. A total of 737 residents aged 60 years or more were enrolled in this study, and the prevalence of hearing impairment was 60.7%. After adjusting for sex, age, education, income, smoking, drinking, systolic blood pressure (SBP), total cholesterol (TC), and low-density lipoprotein cholesterol level (LDL-C), MMSE score and immediate recall score were negatively associated with overall PTA (OPTA) at four frequencies (0.5, 1, 2, and 4 kHz), PTA at low frequencies (LPTA; 0.5, 1, and 2 kHz), and PTA at high frequencies (HPTA; 3, 4, and 8 kHz) in the ear with better hearing. Moreover, orientation score was negatively associated with OPTA and LPTA, and the attention and calculation scores were negatively associated with OPTA and HPTA. Each 10-dB increase in OPTA was associated with a MMSE score decrease of 0.464. Each 10-dB increase in LPTA or HPTA was associated with a MMSE score decrease of 0.441 (95% CI: −0.795, −0.086) and 0.351 (95% CI: −0.592, −0.110), respectively. The present study demonstrated significant but weak relationships between OPTA, LPTA, and HPTA with global cognitive function, as defined using MMSE scores; these relationships were independent of age, education, lifestyle factors, and laboratory test values. These results indicated that hearing was associated with cognitive decline among older individuals, who should be screened routinely to identify risk for cognitive decline.

## Introduction

Cognitive impairment is the leading cause of disability and a global public health priority for aging populations (Wortmann, 2012; GBD 2017 DALYs and HALE Collaborators, 2018). The World Alzheimer Report estimated there were over 50 million people living with dementia globally, and this number was estimated to increase to more than 152 million by 2050 (Wimo et al., 2013). Moreover, most people with dementia live in developing countries, and the number of people living with dementia in China accounts for approximately 25% of total dementia population worldwide, posing a substantial economic and social burden (GBD 2016 Neurology Collaborators, 2019).

Hearing impairment is the third most prevalent chronic condition in older age (Yueh et al., 2003). According to the Global Burden of Disease study, there were 1.4 billion people living with hearing impairment in 2017, and approximately 90% of people with moderate to severe hearing impairment reside in developing countries (GBD 2017 Disease and Injury Incidence and Prevalence Collaborators, 2018). Hearing impairment has also been recognized as the largest potentially modifiable risk factor for dementia and cognitive decline (Livingston et al., 2017; Loughrey et al., 2018). Numerous epidemiological studies have demonstrated hearing impairment at older age was associated with cognitive decline (Lin et al., 2013; Gurgel et al., 2014; Deal et al., 2017; Pabst et al., 2021; Saji et al., 2021). For example, the Health, Aging and Body Composition Study found that hearing impairment at baseline was related to a 24% increased risk of dementia over a 6-year period (Lin et al., 2013). Hearing loss at older age usually impacts high-frequency hearing long before low-frequency hearing (Panza et al., 2015). Thus, it is important to determine whether hearing loss at high frequencies is associated with impaired cognitive function. However, studies exploring significant association between hearing acuity and cognition have used the pure tone average (PTA) threshold at overall or low frequencies (Bush et al., 2015; Golub et al., 2020). To our knowledge, only one cross-sectional study enrolling 307 elderly demonstrated PTA at low frequencies, but not high frequencies, was related to cognitive performance among older individuals (Mukari et al., 2017). The association between PTA at different frequencies and cognitive function is not as well established.

Moreover, socioeconomic inequality is related to the risk of hearing loss (Emmett and Francis, 2015; Ping et al., 2018), and low income is associated with poor auditory function, with approximately 90% residents living in low- and middle-income countries having moderate to profound hearing impairment (Davis and Hoffman, 2019). There is a high burden of hearing impairment in northern China, where the prevalence of hearing impairment is 49.3% among the low-income rural population aged over 45 years (Yang et al., 2021). Only four studies in China have focused on the mediating role of social isolation, cognitive reserve, and leisure activities between self-reported hearing impairment and cognitive decline (Chen and Lu, 2019; Chen and Zhou, 2020; Gao et al., 2020; Chen, 2021). However, the relationship between cognitive performance and PTA at different frequencies as measured using standardized audiometric tests in an older population has not been reported in China, especially in low-income rural areas.

Thus, the aim of this study was to evaluate the relationships between cognitive function and hearing acuity at different frequencies among a low-income elderly population in northern rural China.

## Materials and Methods

### Study Population

This population-based, cross-sectional study recruited older individuals from 18 administrative villages in rural areas of Tianjin, China from April 2012 to November 2013 based on the Tianjin Brain Study (Wang et al., 2014; Hu et al., 2016). Owing to the national health policy, all residents aged 60 years and older visit the health center for free physical examinations annually. From this population, all older residents (≥ 60 years old) with previous diagnosis of total hearing loss (over 120 dB) and blindness (best-corrected distance visual acuity < 3/60 or visual field < 10 central degrees) in the better ear/eye were excluded (Martin, 1986; World Health Organization (WHO), 2020).

The study was approved by the ethics committee at the Tianjin Medical University General Hospital, and written informed consent was obtained from all participants.

### Risk Factors and Physical Examinations

This study was conducted through face-to-face interviews by trained researchers. Demographic information (including name, sex, date of birth, income, and educational level), individual medical history (including the presence of hypertension, diabetes mellitus, stroke, and coronary heart disease), and lifestyle factors (including smoking, drinking, and exercise) were collected using a pre-designed questionnaire; data regarding exercise was missing for six individuals. The participants were categorized into three age groups (60–64, 65–69, and ≥ 70 years), three educational groups (0–5, 6–8, and ≥ 9 years), and three groups of annual per capita income (< 300 USD, 300–650 USD, and > 650 USD). Smoking was defined as smoking ≥ 1 cigarette daily for more than 1 year. Drinking was defined as drinking > 50 mL of alcohol at least once per week for more than 6 months.

Body height, weight, waist circumference, systolic blood pressure (SBP), and diastolic blood pressure (DBP) were obtained by local general practitioners with the participant wearing thin clothing. The levels of fasting blood glucose (FBG), total cholesterol (TC), triglycerides (TG), high-density lipoprotein cholesterol (HDL-C), and low-density lipoprotein cholesterol (LDL-C) were tested in the central laboratory of the Tianjin Ji County People’s Hospital. Hypertension was defined as a SBP ≥ 140 mm Hg, DBP ≥ 90 mm Hg, the use of antihypertensive drugs, or a history of hypertension. Diabetes was defined as a FBG ≥ 7.0 mmol/L, taking medication for diabetes, or a self-reported history of diabetes. Body mass index (BMI) was calculated as the individual’s weight (kg) divided by the square of the individual’s height (m^2^) and was classified into four categories (low-weight, < 18.5 kg/m^2^; normal, 18.5–23.9 kg/m^2^; overweight, 24.0–27.9 kg/m^2^; and obese, ≥ 28.0 kg/m^2^; Zhou, 2002).

### Cognitive Impairment

Cognitive function was measured using the Chinese version of the Mini-Mental State Examination (MMSE) owing to its high sensitivity and specificity of screening for cognitive impairment (Li et al., 1989; Canadian Task Force on Preventive Health Care et al., 2016). The diagnostic criteria of cognitive impairment were based on MMSE score according to educational levels. The MMSE is a 30-point scale that assesses five different cognitive domains including orientation, immediate recall, attention and calculation, recall, and language. Cognitive impairment was defined as an MMSE score < 17 points in the illiterate group, < 22 points in the primary school group, and < 26 points in the junior school and above group (Nunes et al., 2010).

### Hearing Test

Audiometric assessments in each ear were performed at seven frequencies (0.5, 1, 2, 3, 4, 6, and 8 kHz) in a quiet, soundproof room using the Denmark XETA Audiometer (Xeta EN60645-1 type:3 REF:8-04-12207 GN Otometrics A/S Hoerskaetten 92360 Taastrup DENMARK) and TDH 50P transducer (Telephonics, Huntington, NY). Audiometric thresholds were measured at 5-dB increments in decibels of hearing level (dB HL). Outcome variables reported in this study were overall PTA (OPTA) at four frequencies (0.5, 1, 2, and 4 kHz), PTA at low frequencies (LPTA; 0.5, 1, and 2 kHz), and PTA at high frequencies (HPTA; 3, 4, and 8 kHz) in the ear with better hearing. Hearing impairment was defined as OPTA > 25 dB of the better ear according to the World Health Organization’s definition of impairment [World Health Organization (WHO), 1997]. Participants suspected of having hearing impairment were referred to audiologists for final diagnoses.

### Statistical Analysis

Continuous variables (age, BMI, waist circumference, SBP, DBP, FBG, TC, TG, LDL-C, HDL-C, and PTA) are described as means and standard deviations. Categorical variables (binary variables: sex, smoking, drinking, physical exercise, hypertension, diabetes, stroke, hearing impairment, and MMSE group; multi-categorical variables: age, education, income, and BMI groups) are presented as numbers with frequencies. The Student *t*-test was used to compare MMSE score differences between binary variables; the ANOVA test was used to compare MMSE score differences between multi-categorical variables. Univariate linear analyses were used to evaluate the relationship between each continuous variable and the MMSE score. Multiple linear regression analyses were used to evaluate the relationship between PTA and MMSE score after adjusting for independent variables that were statistically significant in the univariate analyses. The univariate analysis results are shown as unadjusted β-values and 95% confidence intervals (CIs); the multivariate analysis results are shown as adjusted β-values and 95% CIs after adjusting for covariates.

All statistical analyses were performed with SPSS version 19.0 statistical software (SPSS Inc., Chicago, IL, United States), and a two-sided *P*-value ≤ 0.05 was considered statistically significant.

## Results

### Demographic Characteristics

A total of 737 residents aged more than 60 years (mean age, 68.95 years) were enrolled in this study, including 324 men (44.0%; mean age, 69.65 years) and 413 women (56.0%; mean age, 68.39 years). In this rural population, the prevalence of hearing impairment was 60.7% overall, 64.2% in men, and 57.9% in women. The mean OPTA of all residents was 30.27 dB HL, with 28.99 dB in LPTA, and 35.44 dB in HPTA. The average education level of the participants was low: 41.7% were illiterate. Moreover, 733 (99.5%) participants had annual per capita incomes of < 650 USD (Table 1).

**TABLE 1:** Demographic characteristics and risk factors for all participants by sex group.

**Risk factors**	**Men**	**Women**	**Total**
Total, *n* (%)	324 (44.0)	413 (56.0)	737 (100.0)
**Age group, *n* (%)**			
60–64 years	84 (25.9)	133 (32.2)	217 (29.4)
65–69 years	97 (29.9)	130 (31.5)	227 (30.8)
70–74 years	63 (19.4)	77 (18.6)	140 (19.0)
≥ 75 years	80 (24.7)	73 (17.7)	153 (20.8)
**Education, *n* (%)**			
0–6 years	57 (17.6)	250 (60.5)	307 (41.7)
6–8 years	190 (58.6)	125 (30.3)	315 (42.7)
≥ 9 years	77 (23.8)	38 (9.2)	115 (15.6)
**Annual per-capital income**			
< 300 USD	262 (80.9)	392 (94.9)	654 (88.7)
300–650 USD	60 (18.5)	19 (4.6)	79 (10.7)
> 650 USD	2 (0.6)	2 (0.5)	4 (0.5)
**Smoking, *n* (%)**			
No	181 (55.9)	386 (93.5)	567 (76.9)
Yes	143 (44.1)	27 (6.5)	170 (23.1)
**Drinking, *n* (%)**			
No	194 (59.9)	397 (96.1)	591 (80.2)
Yes	130 (40.1)	16 (3.9)	146 (19.8)
**Physical exercise, *n* (%)**			
No	231 (72.9)	295 (73.0)	526 (73.0)
Yes	86 (27.1)	109 (27.0)	195 (27.0)
**Hypertension, *n* (%)**			
No	174 (53.7)	214 (51.8)	388 (52.6)
Yes	150 (46.3)	199 (48.2)	349 (47.4)
**Diabetes, *n* (%)**			
No	296 (91.4)	361 (87.4)	657 (89.1)
Yes	28 (8.6)	52 (12.6)	80 (10.9)
**CHD, *n* (%)**			
No	271 (83.6)	330 (79.9)	601 (81.5)
Yes	53 (16.4)	83 (20.1)	136 (18.5)
**Stroke, *n* (%)**			
No	311 (96.0)	396 (95.9)	707 (95.9)
Yes	13 (4.0)	17 (4.1)	30 (4.1)
**Hearing impairment, *n* (%)**			
No	116 (35.8)	174 (42.1)	290 (39.3)
Yes	208 (64.2)	239 (57.9)	447 (60.7)
**BMI category, *n* (%)**			
Low-weight	13 (4.0)	9 (2.2)	22 (3.0)
Normal	150 (46.3)	178 (43.1)	328 (44.5)
Overweight	120 (37.0)	161 (39.0)	281 (38.1)
Obese	41 (12.7)	65 (15.7)	106 (14.4)
**MMSE group, *n* (%)**			
Non-CI	59 (18.2)	31 (7.5)	90 (12.2)
CI	265 (81.8)	382 (92.5)	647 (87.8)
Age, means (SD)	69.65 (6.45)	68.39 (6.10)	68.95 (6.28)
BMI (kg/m^2^), mean (SD)	24.10 (3.31)	24.66 (3.51)	24.42 (3.43)
Waist (cm), mean (SD)	86.90 (9.65)	86.53 (9.22)	86.69 (9.41)
SBP, mean (SD)	5.48 (1.24)	5.55 (1.35)	5.52 (1.30)
DBP, mean (SD)	4.34 (0.86)	4.89 (0.94)	4.65 (0.94)
FBG, mean (SD)	1.19 (0.72)	1.56 (0.90)	1.40 (0.84)
TC, mean (SD)	1.36 (0.43)	1.43 (0.53)	1.40 (0.49)
TG, mean (SD)	2.27 (0.98)	2.74 (1.00)	2.53 (1.02)
HDL-C, mean (SD)	157.85 (23.80)	159.76 (23.83)	158.92 (23.82)
LDL-C, mean (SD)	92.38 (12.28)	91.44 (12.62)	91.85 (12.47)
MMSE score, mean (SD)	22.45 (4.55)	19.18 (5.31)	20.62 (5.24)
OPTA, mean (SD), dB HL	31.30 (10.54)	29.46 (8.59)	30.27 (9.54)
LPTA, mean (SD), dB HL	29.07 (9.44)	28.91 (8.48)	28.99 (8.91)
HPTA, mean (SD), dB HL	38.69 (15.61)	32.87 (11.61)	35.43 (13.81)

*SD, standard deviation; CHD, coronary heart disease; BMI, body mass index; MMSE, Mini-Mental state examination; CI: cognitive impairment; SBP, systolic blood pressure; DBP, diastolic blood pressure; FBG, fasting blood glucose; TC, total cholesterol; TG, triglycerides; HDL-C, high-density lipoprotein cholesterol; LDL-C, low-density lipoprotein cholesterol; OPTA, overall pure tone average; LPTA, low-frequency pure tone average; HPTA, high-frequency pure tone average.*

### Factors Associated With MMSE Score in the Univariate Analysis

The MMSE score was higher among participants with female sex, older age, high education, high income, smoking, and drinking, compared with other groups (all, *P* < 0.001). Compared with individuals with normal hearing, MMSE score of those with hearing impairment did not approached statistical significance (*P* = 0.872; Table 2).

**TABLE 2:** Differences in mean MMSE score, according to demographic characteristics and risk factors groups.

**Characteristics**	**Mean MMSE**	***F/t***	***P***
**Sex**		9.015	< 0.001
Men	22.45 (4.55)		
Women	19.18 (5.31)		
**Age group**		21.077	< 0.001
60–64 years	21.64 (4.63)		
65–69 years	21.70 (4.78)		
70–74 years	20.21 (5.28)		
≥ 75 years	17.94 (5.71)		
**Education**		138.452	< 0.001
0–5 years	17.54 (4.98)		
6–8 years	22.13 (4.38)		
≥ 9 years	24.70 (3.09)		
**Annual per-capital income**		10.266	< 0.001
< 300 USD	20.31 (5.31)		
300–650 USD	22.95 (3.95)		
> 650 USD	24.50 (3.11)		
**Smoking**		−4.224	< 0.001
No	20.20 (5.33)		
Yes	22.00 (4.72)		
**Drinking**		−5.347	< 0.001
No	20.15 (5.30)		
Yes	22.49 (4.58)		
**Hypertension**		−0.317	0.751
No	20.56 (5.23)		
Yes	20.68 (5.27)		
**Diabetes**		−0.082	0.935
No	20.61 (5.25)		
Yes	20.66 (5.23)		
**CHD**		−1.438	0.152
No	20.49 (5.32)		
Yes	21.17 (4.87)		
**Stroke**		−0.728	0.462
No	20.59 (5.25)		
Yes	21.30 (5.12)		
**Hearing impairment**		0.162	0.872
No	20.66 (4.91)		
Yes	20.59 (5.45)		

*CHD, coronary heart disease.*

In the linear regression analysis, MMSE score was negatively associated with SBP, TC, and LDL-C (all, *P* < 0.05; Table 3).

**TABLE 3:** Association of MMSE score with measured parameters in the linear regression analysis.

**Characteristics**	**β (95% CI)**	***P***
SBP	−0.020 (−0.036, −0.004)	0.012
DBP	0.020 (−0.010, 0.050)	0.197
FBG	0.042 (−0.250, 0.333)	0.779
TC	−0.762 (−1.160, −0.363)	<0.001
TG	−0.211 (−0.663, 0.241)	0.359
HDL-C	−0.635 (−.411, 0.140)	0.108
LDL-C	−0.611 (−0.982, −0.241)	0.001

*SBP, systolic blood pressure; DBP, diastolic blood pressure; FBG: fasting blood glucose; TC, total cholesterol; TG, triglycerides; HDL-C, high-density lipoprotein cholesterol; LDL-C, low-density lipoprotein cholesterol.*

### Association of MMSE Score and Its Domains With PTA in the Univariate Analysis

Linear regression analysis showed that MMSE score and its domains, immediate recall, and attention and calculation, were negatively correlated with OPTA, LPTA, and HPTA (all, *P* < 0.05). Orientation was negatively correlated with OPTA and LPTA in the univariate analysis (Table 4).

**TABLE 4:** Association of MMSE and its domains with pure tone average in the linear regression analysis.

**Parameters**	**β**	**SE**	**95% CI**	***P***
**OPTA**				
MMSE score	–0.520	0.202	−0.917, −0.124	0.010
Orientation	–0.126	0.057	−0.238, −0.015	0.026
Immediate recall	–0.127	0.035	−0.196, −0.058	<0.001
Attention and calculation	–0.157	0.068	−0.291, −0.023	0.022
Recall	0.072	0.048	−0.022, 0.166	0.133
Language	–0.087	0.079	−0.242, 0.068	0.269
**LPTA**				
MMSE score	–0.601	0.216	−1.025, −0.177	0.006
Orientation	–0.157	0.061	−0.276, −0.038	0.010
Immediate recall	–0.129	0.038	−0.203, −0.055	0.001
Attention and calculation	–0.155	0.073	−0.298, −0.011	0.035
Recall	0.089	0.051	−0.011, 0.189	0.082
Language	–0.137	0.084	−0.303, 0.028	0.104
**HPTA**				
MMSE score	–0.347	0.140	−0.621, −0.073	0.013
Orientation	–0.065	0.039	−0.142, 0.012	0.096
Immediate recall	–0.092	0.024	−0.140, −0.044	<0.001
Attention and calculation	–0.130	0.047	−0.222, −0.037	0.006
Recall	0.023	0.033	−0.042, 0.088	0.695
Language	–0.034	0.054	−0.141, 0.073	0.532

β, *regression coefficient; SE, standard error; CI, confidence interval; OPTA, overall pure tone audiometry; LPTA, low-frequency pure tone average; HPTA, high-frequency pure tone average.*

### Association of MMSE Score and Its Domains With PTA in the Multiple Linear Regression Analysis

Mini-Mental State Examination score and immediate recall score were negatively associated with OPTA, LPTA, and HPTA in multiple linear regression analyses after adjusting for sex, age, education, income, smoking, drinking, SBP, TC, and LDL-C (all, *P* < 0.05). Moreover, orientation score was negatively associated with OPTA and LPTA (all, *P* < 0.05), and attention and calculation score were negatively associated with OPTA and HPTA after adjusting for sex, age, education, income, smoking, drinking, SBP, TC, and LDL-C (all, *P* < 0.05; Table 5). The *R*^2^ value in linear regression was similar between LPTA and HPTA (adjusted *R*^2^ 0.332 vs. 0.334).

**TABLE 5:** Association of MMSE and its domains with pure tone average in the multiple linear analysis.

**Parameters**	**β (95% CI)**		
	**OPTA**	**LPTA**	**HPTA**
MMSE score	−0.464 (−0.798, −0.130)**	−0.441 (−0.795, −0.086)*	−0.351 (−0.592, −0.110)**
Orientation	−0.114 (−0.218, −0.009)*	−0.122 (−0.232, −0.012)*	–
Immediate recall	−0.107 (−0.175, −0.040)**	−0.100 (−0.171, −0.028)**	−0.082 (−0.131, −0.034)**
Attention and calculation	−0.130 (−0.253, −0.007)*	−0.101 (−0.232, 0.029)	−0.121 (−0.210, −0.033)**

*P < 0.05. ** *P* < 0.01. Adjusted for sex, age, education, income, smoking, drinking, TC, LDL-C, and SBP.

## Discussion

This study evaluated the associations between peripheral hearing with global cognitive function and five domains of cognition among low-income elderly individuals in northern rural China. The prevalence of hearing impairment was 60.7% in this low-income rural population. LPTA and HPTA were negatively and independently related to MMSE score and its domains; this association was independent of age, education, lifestyle factors, and laboratory test values. In multiple linear regression analysis, both LPTA and HPTA accounted for a minimal proportion variance of MMSE score after adjusting for other covariates. There was a 0.441-point and 0.351-point MMSE score decrease associated with each 10-dB increase in LPTA and HPTA, respectively.

Hearing loss is prevalent among older adults and associated with a high prevalence of cognitive decline, apathy, and poor functional status (Sugawara et al., 2011; Miyake et al., 2020). In the present study, four-frequency PTA in the ear with better hearing was an independent risk factor for global cognitive status (MMSE score) and its domains; these findings are consistent with most previous studies. For instance, a longitudinal community-dwelling study found that hearing loss was related to accelerated cognitive decline and dementia among older adults, and individuals with hearing impairment at baseline was related to a 24% increased risk of dementia after a 6-year follow-up (Lin et al., 2013). Two American epidemiologic studies also found an independent association between cognitive performance and subclinical hearing loss; there was a 0.97-point decrease in the Digit Symbol Substitution Test score associated with a 10-dB increase of the PTA (Golub et al., 2020). Furthermore, a systematic review and meta-analysis demonstrated that hearing impairment was associated with a decline of global cognition, cognitive domains of executive function, and episodic memory, as well as increased risk of incident dementia and cognitive impairment (Loughrey et al., 2018). Other studies found stronger associations between hearing decline and lower episodic memory levels (Maharani et al., 2018b; Guglielmi et al., 2020). As a proxy measurement for episodic memory, immediate recall was strongly associated with worse hearing acuity in present study. Moreover, hearing aids use helps to slow down cognitive decline and improve functional status of older individuals (Maharani et al., 2018a; Sarant et al., 2020). However, a prospective cohort study in four American metropolitan areas demonstrated that vision, but not hearing impairment, was associated with cognitive decline (Lin et al., 2004). Another longitudinal study found that hearing loss did not accelerate cognitive decline over time after adjusting for the non-linear effects of age (Croll et al., 2021). In the present study, PTA was negatively and independently related to MMSE score and its domains independent of age, education, lifestyle factors, and laboratory test values.

Additionally, we found an independent association of both LPTA and HPTA with MMSE score. To our knowledge, only one study has assessed the association between PTA of different frequencies and cognitive performance; this prior study reported that LPTA, but not HPTA, was significantly and independently related to the MMSE score (Mukari et al., 2017). Studies have consistently confirmed that PTA is highly correlated with speech recognition (Coren and Hakstian, 1994; Vermiglio et al., 2012). In addition, a cross-section study demonstrated that PTA at low frequency exhibited the highest effect on speech recognition threshold compared to PTA at full range and high frequencies (Coren and Hakstian, 1994). High LPTA was more associated with poor speech recognition, which resulted in difficulty in communicating and maintaining interpersonal relationships (Lindenberger and Baltes, 1994; Maharani et al., 2019). These reasons will further cause social isolation, loneliness, and cognitive decline.

Three hypotheses have been proposed to explain the association between hearing and cognitive function (Wayne and Johnsrude, 2015; Uchida et al., 2019). In the cognitive load hypothesis, auditory signals are degraded among individuals with hearing loss (Lavie, 1995). Consequently, greater cognitive resource is required to understand speech, which affects other cognitive tasks and results in cognitive reserve depletion (Tun et al., 2009). Excessive cognitive load in daily life would cause neurodegeneration and structural changes in the brain, which subsequently impairs cognitive function (Martini et al., 2014). In addition, according to the common cause hypothesis, hearing impairment usually occurs simultaneously with cognitive decline at older ages; both hearing impairment and cognitive decline are results of neuropathological cause without direction of causality (Stahl, 2017). Finally, the sensory deprivation hypothesis suggests that sensory impairment, like hearing and vision impairment, could prevent older adults from communicating, resulting in social isolation, loneliness, and poor cognitive status (Rutherford et al., 2018). Some studies have reported the mediating effect of social isolation and loneliness between hearing and cognition (Rutherford et al., 2018; Maharani et al., 2019).

This was a population-based real-world study. Although studies have shown that hearing impairment increases the risk of cognitive decline, the relationship between hearing acuity and cognition remains inconclusive, especially in studies of large-scale low-income people. Moreover, many factors including age, sex, education, income, blood pressure, serum lipids, diabetes, smoking, and drinking can affect cognitive function (Yaffe et al., 2021). In the present study, both LPTA and HPTA accounted for a minimal proportion variance of the MMSE score; this association was independent of age, education, lifestyle factors, and laboratory test values. Moreover, due to earlier hearing loss at high frequencies, it is of great importance to discover and manage hearing loss to reduce risk for cognitive decline on the early stage.

There are several limitations in this study. First, cognitive function was evaluated using MMSE scores rather than a cognitive test battery; therefore, cognitive domain deficit could not be further diagnosed. Second, the speech-in-noise test could better simulate communication environments of daily living. The Mandarin Quick Speech-in-Noise test (M-Quick SIN) is quick and reliable with high clinical feasibility (Zhou et al., 2014) in population-based study. As M-Quick SIN was not established until 2014, it was not included in the present study. In the future, we plan to conduct a study including the speech-in-noise test. Third, the study population was from a low-income, low-education, rural population in northern China, thus its representativeness and generalizability are limited. Fourth, other confounding factors, including APOE4 genotype and diet, are important factors for cognitive decline and were not excluded in this study (Davies et al., 2018; Kivipelto et al., 2018). Fifth, asymmetrical hearing can be detrimental to cognitive function (Brännström et al., 2018) but was not included in the present study. Our follow-up research will further focus on asymmetrical hearing. Last, this was a cross-sectional study, and therefore causal relationships could not be identified.

## Conclusion

The present study demonstrated significant but weak relationships between OPTA, LPTA, and HPTA with global cognitive function, as defined using MMSE scores, independent of age, education, lifestyle factors, and laboratory test values. These results indicate that hearing was associated with cognitive decline among older individuals, who should be screened routinely to identify risk for cognitive decline.

## Data Availability Statement

The raw data supporting the conclusions of this article will be made available by the authors, without undue reservation.

## Ethics Statement

The studies involving human participants were reviewed and approved by The Ethics Committee of Tianjin Medical University General Hospital. The patients/participants provided their written informed consent to participate in this study.

## Author Contributions

DY, XL, and XN were involved in the conception and design of the study, data interpretation, and critically reviewed the manuscript. YX, YL, DG, XZ, HG, HC, XL, JZ, JT, and DY were involved in the data collection, case diagnosis, and confirmation for this manuscript. YX, YL, DG, and XZ were involved in the manuscript drafting and revision. JW was involved in the data analysis for this manuscript. All authors contributed to the article and approved the submitted version.

## Conflict of Interest

The authors declare that the research was conducted in the absence of any commercial or financial relationships that could be construed as a potential conflict of interest.

## Publisher’s Note

All claims expressed in this article are solely those of the authors and do not necessarily represent those of their affiliated organizations, or those of the publisher, the editors and the reviewers. Any product that may be evaluated in this article, or claim that may be made by its manufacturer, is not guaranteed or endorsed by the publisher.
